# Association of Job Characteristics and Burnout of Healthcare Workers in Different Positions in Rural China: A Cross-Sectional Study

**DOI:** 10.3389/ijph.2023.1605966

**Published:** 2023-08-03

**Authors:** Mei Zhang, Sangsang Li, Dan Han, Yunyi Wu, Jie Zhao, Hui Liao, Ying Ma, Chaoyang Yan, Jing Wang

**Affiliations:** ^1^ Department of Health Management, School of Medicine and Health Management, Tongji Medical College, Huazhong University of Science and Technology, Wuhan, Hubei, China; ^2^ The Key Research Institute of Humanities and Social Science of Hubei Province, Huazhong University of Science and Technology, Wuhan, Hubei, China; ^3^ Institute for Poverty Reduction and Development, Huazhong University of Science and Technology, Wuhan, Hubei, China

**Keywords:** occupational stress, burnout, job characteristic, rural healthcare worker, different position

## Abstract

**Objectives:** Health workers in rural primary care systems are at increased risk of job burnout, but their associations with different positions have received scant attention in the literature. Thus, this study aims to measure job burnout in different positions in rural China and to identify factors associated with it.

**Methods:** A cross-sectional survey was conducted with a total of 15,627 participants from six provinces in China. And job burnout was measured using the Chinese version of the Maslach Burnout Inventory-General Scale (MBI-GS). Multilevel regression analyses were used in examining factors potentially associated with job burnout in different positions.

**Results:** Overall, more than half of providers suffered from moderate burnout. The degree of job burnout varied among different positions. Middle managers showed higher levels personal stress, while general staff showed the lowest interpersonal and self-evaluation dimensions of burnout. Job duty, job capability, job treatment, and career advancement are potential factors affecting these results.

**Conclusion:** Interventions aimed at providing appropriate training and development opportunities, developing relevant career planning and management strategies, and implementing reasonable staffing and job design may be promising strategies for alleviating burnout in different positions and improving health system performance.

## Introduction

Burnout is a reaction to the imbalance between work-related demands and personal resources, and it is manifested through the three dimensions: emotional exhaustion (EE), depersonalization (DP), and reduced personal accomplishment (PA) [1, 2], which represent basic personal stress, interpersonal environment, and the self-evaluation dimensions of burnout, respectively [3]. Healthcare professionals often suffer from burnout [4, 5]. According to a systematic review published in 2018, the prevalence rates of EE, DP, and reduced PA were 27.4%–99.6%, 13.3%–98.0%, and 25.1%–99.3%, respectively, in primary care (PC) providers in low- and middle-income countries [6]. High job burnout is frequent among PC providers in China [4]. A national survey conducted for 10,626 primary healthcare doctors revealed that 41% of the respondents felt highly exhausted; 37%, highly depersonalized; and 34%, a low sense of PA.

Burnout can have many negative effects on an individual and organization. Long-term burnout can damage an individual’s physical and mental health [7], and burnout in health professionals is related to low job commitment [8], high turnover intention [9–11], and poor work performance [12–15]. Provider job burnout is associated with adverse events, including medical errors, reduction in quality of care [16, 17], and poor patient satisfaction [18, 19], and job burnout is affected by many factors.

In general, job burnout is associated with socioeconomic status. Men are more likely to suffer from job burnout than women [5, 20], and age is associated with job burnout symptoms [21]. Compared with married people, unmarried people have higher levels of job burnout. Moreover, high levels of education are risk factors for job burnout [22], and job burnout rate in medical staff in the eastern region is higher than the job burnout rates in the central and western regions.

Job characteristics, including professional title, professional status, and working hours per week, can be tightly related to job burnout. These job characteristics can help us understand the relationship between specialization levels, responsibilities, role positioning, workload, and burnout across different practice types. A survey in Turkey published in 2021 found that burnout level decreased with increasing academic title, and attending physicians were the most exhausted [23]. Working over 40 h per week was the most important risk factor for burnout in PC physicians in Oman [24]. In rural China, primary healthcare workers with middle- or high-ranking professional titles had high degrees of job burnout [25]. Compared with doctors and nurses, pharmacists suffer a higher degree of job burnout. Working for over 40 h per week is related to high levels of burnout.

However, previous studies on the job burnout in medical staff in primary health facilities focused on burnout in different types of health technicians (physicians, nurses, community health workers, midwives, and pharmacists) and its influencing factors. However, this study places greater emphasis on exploring the differences in job burnout among individuals in different positions within the same institution. On the one hand, as a populous country, China faces challenges of uneven distribution of primary healthcare human resources, with rural areas experiencing shortages in meeting primary healthcare service demands. The supply-demand imbalance of primary healthcare services is a common issue in other developing countries around the world. On the other hand, China has a large population, and there is a significant disparity in medical resources between urban and rural areas, with generally low capacity for primary medical services. In addition to the need for rational allocation of medical resources, improving management efficiency is also crucial. Therefore, unlike developed countries with relatively abundant medical resources or other developing countries with less pressure on primary healthcare issues, conducting this study within the context of China has unique value.

The present study focused on job burnout in primary health facilities in China, determined the deep possible reasons of different positions, especially administrative positions. The main objective is to identify the underlying causes, particularly in administrative roles, and explore targeted intervention measures. Hence, the aims of this study were 1) to measure the prevalence of job burnout in different managers in China and 2) to identify the associated factors of job burnout for different positions.

## Methods

### Data Source and Sample Selection

**FIGURE 1:**
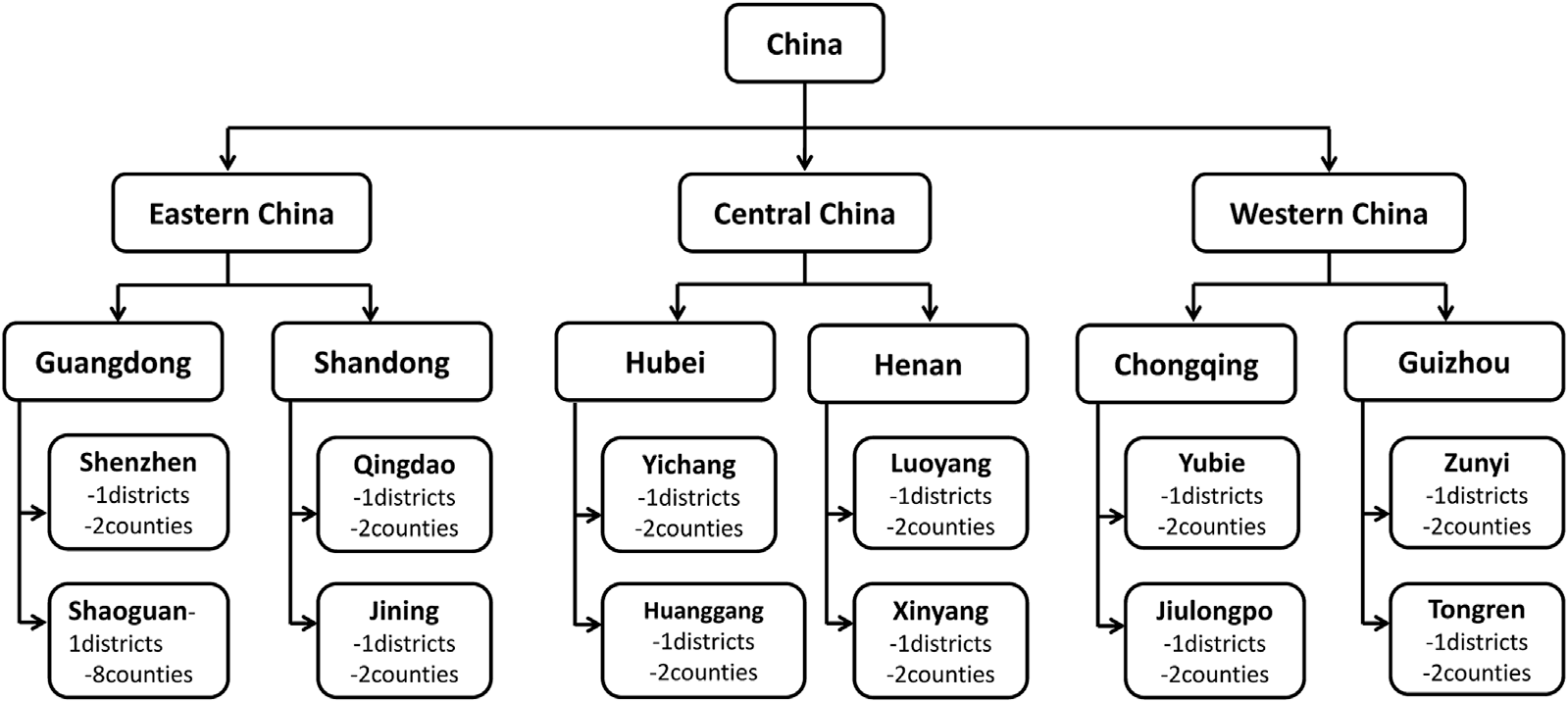
A multistage stratified cluster sampling (China, 2022).

A cross-sectional study was conducted in six provincial regions in China from October 2018 to November 2018. Sampled was performed at the provincial–municipal–district/county level through three stages. First, six provincial-level regions (Shandong, Guangdong, Henan, Hubei, Guizhou, and Chongqing) were selected according to socioeconomic level and geographical distribution. Then, one developed city and one less-developed city were randomly selected from each of the six provinces according to gross regional product *per capita*. One district and two counties were randomly selected from the 12 cities. At the request of the local health bureau, we conducted a survey on the remaining six PC facilities that were not sampled in Shaoguan City. Finally, 42 districts/counties were included (Figure 1) [26, 27].

**FIGURE 2:**
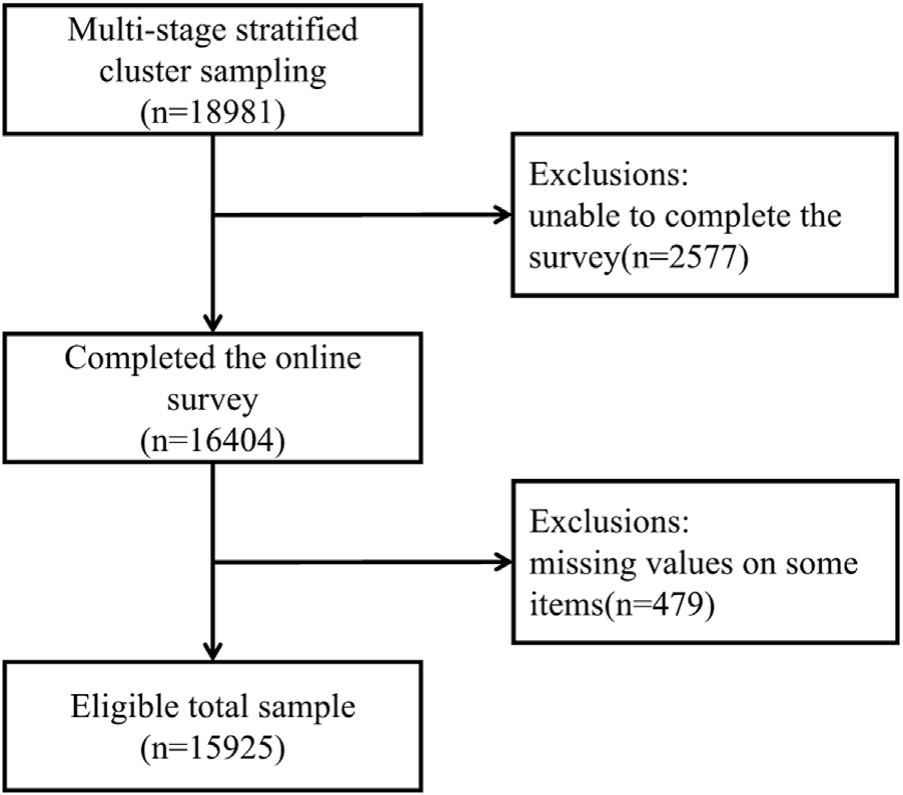
Flowchart of sample selection (China, 2022).

Data were collected through an online survey. To encompass healthcare professionals with diverse job characteristics, we conducted an online questionnaire survey of all healthcare professionals in the surveyed healthcare institutions, ensuring, the inclusion of the entire population in our study. With the assistance of the local health authorities, the research team established online communication with the local township hospitals and had the leaders of each township health center forward the online questionnaire on our behalf. The main questionnaire of this study contained information: basic demographic information, occupational information, and burnout information. All the participants provided consent to participate, and de-identification and confidentiality were ensured before they answered the questionnaires. A total of 16,404 PC providers participated in the survey, and the response rate was 86.42% (16,404 out of 18,981). Cases with missing values in the surveys or with wrong information were excluded. The final sample size was 15,925 PC providers, and the effective response rate was 83.90% (Figure 2).

### Variables and Definitions

#### Dependent Variable

The main dependent variable is burnout. Burnout was measured using the Chinese version of the 16-item Maslach Burnout Service Inventory (MBI-GS), which had previously been validated among medical staff in China [28, 29]. The 16-item Chinese version of the Maslach Burnout Inventory-General Scale (MBI-GS) was used [15, 30, 31], which includes EE (five items), DP (five items), and reduced PA (six items). Each item contains a 7-point Likert scale ranging from 0 (“never”) to 6 (“every day”). High scores on EE and DP and low scores on PA indicate severe burnout. Therefore, the six items for PA were reversely scored. Used by several previous studies, the following equation was adopted to produce the weighted sum score of burnout [28, 29, 32]:

Burnout=0.4×EE+0.3×DP+0.3×reduced PA (1)

According to the results of burnout, the participants were divided into three groups: no burnout group (0–1.49), moderate burnout group (1.50–3.49), and severe burnout group (3.5–6.0) [33, 34]. Participants were defined as “burnout” when they were in moderate or severe burnout.

#### Independent Variables

The main independent variables were job characteristics. The job characteristics included professional status (doctors/nurse/pharmacist/others), the rank of professional title (primary/medium/high), positions (senior managers/middle managers/general staff), and workload (<40/40–49/50–59/60–69/≥70 h per week). Laboratory technicians and medical imaging specialists were included in the category of doctors.

#### Control Variables

The Socioeconomic Status (SES) variables included gender (male/female), education level (junior school/high school/university), age (≤29/30–39/40–49/≥50 years old), marital status (married/unmarried), and the location of facility (eastern/central/western). The university category included postgraduate, and the unmarried category included single, widowed, divorced, and separated status in the questionnaires.

### Statistical Analysis

Frequency (N) and percentage (%) were used in analyzing the socio-demographic and job characteristics of the participants, and chi-square tests were conducted to determine differences among positions. Formula 1 was used in calculating scores in each dimension and the weighted sum score of job burnout. The chi-square tests were conducted to determine differences among positions. Multilevel linear regression was used in examining the factors of job burnout for senior managers, middle managers, and general staff. In the multiple linear regression analysis of this study, categorical variables such as gender, education level, marital status, and region were first transformed into dummy variables and then incorporated into the regression equation for analysis. All statistical analyses were performed using Stata 13.0. Statistical significance was set at *p* < 0.1 and *p* < 0.05.

## Results

Table 1 shows the characteristics of the participants. The majority of the participants were females (65.49%), and participants aged 50 (7.51%) had the least number. Most of the participants were living with a partner (78.88%). Approximately 69.83% of the participants received low levels of education, and only 3.55% of the participants had high-ranking professional titles. Physicians accounted for 46.98% of the participants surveyed; nurses, 28.20%; others, 17.07%; and pharmacists, 7.76%. Most of the participants were practicing more than 40 h per week (93.16%), and 37.00%, 24.24%, and 38.75% were from eastern, central, and western China, respectively. General staff accounted for 79.83% of the participants surveyed; middle managers, 13.53%; and senior managers, 6.64%. Significant differences in the majority of these characteristics were found among the three types of participants (*p <* 0.05). Overall, the proportion of the burnout cases was 52.01% (48.61% experienced moderate burnout and 3.40% suffered from severe burnout). The proportions of burnout cases in the three groups were 54.69%, 50.81%, and 51.98%. The proportion of serious burnout cases in the three groups were 3.03%, 3.71%, and 3.37%.

**TABLE 1:** General characteristics of the sample participants (*N*, %) (China, 2022).

	Overall (*N* = 15,925)	Senior managers (*N* = 1,057)	Middle managers (*N* = 2,155)	General staff (*N* = 12,713)	*p*
Independent variable					
Gender					
Male	5,424 (34.06)	883 (83.54)	1,150 (53.36)	3,391 (26.67)	<0.001
Female	10,501 (65.49)	174 (16.46)	1,005 (46.64)	9,322 (73.33)	
Age group(years)					
≤29	5,456 (34.26)	33 (3.12)	182 (8.45)	5,241 (41.23)	<0.001
30–39	5,207 (32.70)	327 (30.94)	780 (36.19)	4,100 (32.25)	
40–49	4,066 (25.53)	556 (52.60)	914 (42.41)	2,596 (20.42)	
≥50	1,196 (7.51)	141 (13.34)	279 (12.95)	776 (6.10)	
Marital status					
Married	12,561 (78.88)	1,022 (96.69)	2,023 (93.87)	9,517 (74.86)	<0.001
Unmarried	3,363 (21.12)	35 (3.31)	132 (6.13)	3,196 (25.14)	
Education level					
Junior high Schools	3,076 (19.32)	102 (9.65)	384 (17.82)	2,590 (20.37)	<0.001
High Schools	8,044 (50.51)	439 (41.35)	898 (41.67)	6,707 (52.76)	
Universities	4,805 (30.17)	516 (48.82)	873 (40.51)	3,416 (26.87)	
Professional title					
Primary or below	12,048 (75.65)	528 (49.95)	1,162 (53.92)	10,358 (81.48)	<0.001
Medium	3,311 (20.79)	381 (36.05)	821 (38.10)	2,110 (16.60)	
High	565 (3.55)	148 (14.00)	172 (7.98)	245 (1.93)	
Professional status					
Physicians	7,481 (46.98)	844 (79.85)	1,143 (53.04)	5,494 (43.22)	<0.001
Nurses	4,491 (28.20)	52 (4.92)	349 (16.19)	4,090 (32.17)	
Pharmacists	1,235 (7.76)	35 (3.31)	242 (11.23)	958 (7.54)	
Others	2,718 (17.07)	126 (11.92)	421 (19.54)	2,171 (17.08)	
Working hours(per week)					
<40	1,089 (6.84)	38 (3.60)	103 (4.78)	948 (7.46)	<0.001
40–49	7,023 (44.10)	445 (42.10)	1,036 (48.07)	5,542 (43.59)	
50–59	3,889 (24.42)	299 (28.29)	516 (23.94)	3,074 (24.18)	
60–69	1,731 (10.87)	146 (13.81)	216 (10.02)	1,369 (10.77)	
≥70	2,193 (13.77)	129 (12.20)	284 (13.18)	1,780 (14.00)	
Location					
Eastern	5,893 (37.00)	310 (29.33)	950 (44.08)	4,633 (36.44)	<0.001
Central	3,861 (24.24)	262 (24.79)	568 (26.36)	3,031 (23.84)	
Western	6,171 (38.75)	485 (45.88)	637 (29.56)	5,049 (39.72)	
Dependent variable					
Job burnout					
No burnout symptoms	7,643 (47.99)	479 (45.32)	1,060 (49.19)	6,104 (48.01)	0.176
Some burnout symptoms	7,741 (48.61)	546 (51.66)	1,015 (47.10)	6,180 (48.61)	
Serious burnout symptoms	541 (3.40)	32 (3.03)	80 (3.71)	429 (3.37)	

*p* value of Chi-square test to examine the significance of the difference in the variable between senior managers, middle managers, and the general staff.

Table 2 shows the scores in each dimension and the weighted sum score of job burnout. The mean score for burnout among the respondents was 1.60, and the median was 1.55, which met the criterion of moderate burnout. The comparison among the dimensions of burnout indicated that the degree of EE was relatively higher than DP and reduced PA in the current sample. The results of t-tests showed no difference in the weighted sum score of job burnout but indicated a significant differences in each dimension of job burnout among the three types of participants. Compared with senior managers and general staff, middle managers had higher levels of EE (2.13, 2.08, and 1.88) and lower levels of reduced PA (1.48, 1.73, and 1.80). Compared with senior and middle managers, the general staff had higher levels of DP (1.03, 0.91, and 1.00), higher levels of reduced PA (1.80, 1.73, and 1.48), and lower levels of EE (1.88, 2.08, and 2.13). Compared with middle managers and general staff, senior managers had lower levels of DP (0.93, 1.00, and 1.03).

**TABLE 2:** Scores in each dimension and the weighted sum score of job burnout (China, 2022).

	Overall (*N* = 15,925)		Senior managers (*N* = 1,057)		Middle managers (*N* = 2,155)		General staff (*N* = 12,713)		*p* ^*a*^
	Mean ± SD	Median(R)	Mean ± SD	Median(R)	Mean ± SD	Median(R)	Mean ± SD	Median(R)	
Weighted sum score of burnout	1.60 ± 0.96	1.55 (0, 5.84)	1.63 ± 0.95	1.63 (0, 4.83)	1.59 ± 0.97	1.51 (0, 5.84)	1.61 ± 0.96	1.55 (0, 5.80)	0.376
EE (5 items, 0–30)	1.93 ± 1.31	1.80 (0, 6.00)	2.08 ± 1.39	2.00 (0, 6.00)	2.13 ± 1.39	2.00 (0, 6.00)	1.88 ± 1.28	1.80 (0, 6.00)	<0.001
DP (4 items, 0–30)	1.02 ± 1.09	0.75 (0, 6.00)	0.91 ± 1.04	0.50 (0, 6.00)	1.00 ± 1.11	0.75 (0, 6.00)	1.03 ± 1.09	0.75 (0, 6.00)	0.001
PA^b^ (6 items, 0–36)	1.75 ± 1.47	1.50 (0, 6.00)	1.73 ± 1.47	1.50 (0, 6.00)	1.48 ± 1.35	1.17 (0, 6.00)	1.80 ± 1.49	1.67 (0, 6.00)	<0.001

EE, emotional exhaustion; DP, depersonalization; PA, personal accomplishment; SD, standard deviation; R, range.

p^a^ value of Chi-square test to examine the significance of the difference in the variable between senior managers, middle managers, and the general staff.

PA^b^, the scores of items of reduced personal accomplishment were reversed in the analysis of single dimension.

Table 3 shows the results of the multilevel regression analysis of the factors associated with burnout and its three subcomponents among different positions. Health workers with medium-ranking professional titles had higher levels of burnout, EE, and DP in the middle managers and general (*p* < 0.05), whereas those from the senior managers showed no difference (*p* > 0.05). Senior managers had higher levels of reduced PA, whereas middle managers and the general staff showed no difference (*p* > 0.05). Compared with physicians, pharmacists in middle manager and general staff positions showed low degrees of burnout, EE, and DP (*p* < 0.05), whereas pharmacists in senior manager positions, showed no difference (*p* > 0.05). Other types of workers in senior manager positions showed high levels of reduced PA (*p* < 0.05), whereas those in middle manager and general staff positions showed low levels of reduced PA (*p* > 0.05). Workload of no less than 40 h per week was associated with low level of reduced PA in senior manager, middle manager, and general staff positions (*p* > 0.05) but high level of EE (*p* > 0.05).

**TABLE 3:** The result of the multilevel regression analysis of the factors associated with burnout (China, 2022).

	Overall (*N* = 15,925)				Senior Managers (*N* = 1,057)				Middle Managers (*N* = 2,155)				General Staff (*N* = 12,713)			
	Burnout	EE	DP	PA^a^	Burnout	EE	DP	PA^a^	Burnout	EE	DP	PA^a^	Burnout	EE	DP	PA^a^
Constant	1.673**	1.699**	1.145**	2.139**	1.337**	1.260**	0.619**	2.050**	1.780**	1.923**	1.078**	2.242**	1.650**	1.669**	1.136**	2.139**
Gender																
Male	—	—	—	—	—	—	—	—	—	—	—	—	—	—	—	—
Female	−0.073**	−0.098**	−0.055**	−0.054**	−0.007	0.006	−0.027	0.015	−0.100**	−0.091	−0.123**	−0.076	−0.077**	−0.100**	−0.070**	−0.051*
Age group (years)																
≤29	—	—	—	—	—	—	—	—	—	—	—	—	—	—	—	—
30–39	0.008	0.073**	−0.003	−0.069*	0.414**	0.474*	0.237	0.557*	−0.072	−0.110	−0.050	−0.029	−0.002	0.058*	−0.002	−0.084**
40–49	−0.123**	−0.026	−0.160**	−0.218**	0.304	0.183	0.096	0.707**	−0.137	−0.187	−0.115	−0.081	−0.147**	−0.019	−0.174**	−0.293**
≥50	−0.189**	−0.063	−0.209**	−0.340**	0.224	0.253	0.012	0.423	−0.138	0.037	−0.123	−0.368*	−0.266**	−0.206**	−0.267**	−0.349**
Marital status																
Married	—	—	—	—	—	—	—	—	—	—	—	—	—	—	—	—
Unmarried	0.045**	−0.022	0.089**	0.095**	0.236	0.302	0.338*	0.033	−0.038	−0.042	−0.024	−0.021	0.047**	−0.020	0.090**	0.097**
Education level																
Junior Schools	—	—	—	—	—	—	—	—	—	—	—	—	—	—	—	—
High Schools	0.052**	0.130**	0.031	−0.031	0.221**	0.320**	0.200*	0.114	0.181**	0.283**	0.174**	0.057	0.033	0.101**	0.011	−0.036
Universities	0.103**	0.292**	0.130**	−0.169**	0.074	0.258	0.105	−0.172	0.126**	0.311**	0.175**	−0.153	0.119**	0.310**	0.162**	−0.173**
Professional title																
Primary or below	—	—	—	—	—	—	—	—	—	—	—	—	—	—	—	—
Medium	0.075**	0.150**	0.088**	−0.040	0.007	0.076	0.117	−0.184*	0.126**	0.152**	0.168**	0.042	0.076**	0.159**	0.082**	−0.041
High	0.033	0.096*	−0.013	−0.013	0.079	0.111	0.096	0.011	0.206**	0.310**	0.142	0.111	−0.069	−0.060	−0.076	−0.083
Professional status																
Physicians	—	—	—	—	—	—	—	—	—	—	—	—	—	—	—	—
Nurse	−0.098**	−0.137**	−0.067**	−0.077**	−0.208	−0.314	−0.321*	0.013	−0.089	−0.127	−0.066	−0.063	−0.100**	−0.132**	−0.065**	−0.092**
Pharmacist	−0.225**	−0.345**	−0.183**	−0.106**	−0.150	−0.269	−0.214	0.033	−0.191**	−0.301**	−0.203**	−0.034	−0.242**	−0.365**	−0.188**	−0.130**
Others	−0.189**	−0.245**	−0.161**	−0.145**	0.102	−0.109	0.138	0.320**	−0.262**	−0.309**	−0.250**	−0.214**	−0.204**	−0.259**	−0.171**	−0.162**
Working hours(per week)																
<40	—	—	—	—	—	—	—	—	—	—	—	—	—	—	—	—
40–49	−0.001	0.112**	−0.028	−0.125**	−0.123	0.102	0.030	−0.574**	−0.030	0.091	0.021	−0.264*	0.005	0.111**	−0.034	−0.097**
50–59	0.095**	0.295**	0.034	−0.110**	0.007	0.359	0.082	−0.546**	0.069	0.293**	0.091	−0.280**	0.101**	0.289**	0.025	−0.073
60–69	0.166**	0.485**	0.078*	−0.172**	0.072	0.695**	0.098	−0.812**	0.100	0.512**	0.016	−0.405**	0.180**	0.463**	0.093**	−0.108*
≥70	0.278**	0.775**	0.190**	−0.298**	0.440**	1.189**	0.414**	−0.494**	0.144	0.649**	0.077	−0.483**	0.275**	0.751**	0.186**	−0.270**
Location																
Eastern	—	—	—	—	—	—	—	—	—	—	—	—	—	—	—	—
Central	−0.266**	−0.323**	−0.271**	−0.175**	−0.093	−0.128	−0.048	−0.054	−0.269**	−0.367**	−0.260**	−0.123	−0.256**	−0.304**	−0.268**	−0.179**
Western	0.086**	−0.089*	−0.031	0.453**	−0.016	−0.260**	−0.162*	0.486**	0.103	0.007	0.063	0.310**	0.125**	−0.032	0.012	0.457**

EE, emotional exhaustion; DP, depersonalization; PA, personal accomplishment.

PA^a^, the scores of items of reduced personal accomplishment were reversed in the analysis of single dimension.

*p-value < 0.1, **p-value < 0.05.

Female providers in the middle manager and general staff positions showed low levels of burnout (*p* < 0.05), whereas those in senior manager positions showed no difference (*p >* 0.05). Senior managers in the 30–49 age group experienced high levels of reduced PA, whereas providers in general staff positions at the same age group showed lower levels of reduced PA and providers in middle manager positions showed no difference (*p* > 0.05). Unmarried providers in senior manager and general staff positions indicated high levels of DP (*p* < 0.05). High level of education was related to high levels of burnout, EE, and DP in middle managers (*p* < 0.05). By contrast, providers in senior manager positions and with highest level of education (undergraduates) showed no difference from providers with the lowest level of education (junior college or below). Providers in the general staff positions showed lower levels of reduced PA, whereas those in senior manager and middle manager positions showed no difference (*p* > 0.05). Compared with participants from eastern China, participants from central China showed lower levels of burnout, EE, and DP, particularly those in middle manager and general staff positions (*p* < 0.05). Senior managers from central China showed no difference (*p* > 0.05).

## Discussion

This study measured job burnout among different positions in China and identified associated factors of job burnout for different positions. More than half of providers in different positions suffered from moderate burnout. Different levels of job burnout in different positions. Middle managers showed the highest levels of personal stress, and general staff showed the lowest interpersonal and self-evaluation dimensions of burnout. Job duty, job capability, job treatment, and career advancement in different positions may be potential factors for these results.

The job duty of different positions, which can be reflected in the content of work goals and job requirements, can be the main and direct reason for job burnout in different positions. Senior managers of hospitals are concerned about the normal operations and sustainable development of their respective hospitals [34]. Middle managers of hospitals, such as directors of departments, are the implementors of hospitals’ strategic goals and policies. They focus on the realization of development goals, such as department discipline construction, talent training, and medical quality [35–38]. They need to face the dual pressure of realizing hospital strategies and their own development, and thus are more emotionally exhausted than workers in the other two positions. The general staff has more repetitive tasks but less job autonomy [39]. This situation may lead to reduced PA.

Difference in job capability may lead to differences in job burnout. First, a large portion of the time of senior managers is spent on communication. They can handle interpersonal problems well. Thus, the level of DP is lower in these workers. Most middle managers received good professional education and have good professional and communication skills. Therefore, they not only get recognition for diagnosis and treatment activities but also are recognized by senior managers and supported by general staff when conducting management activities. Hence, middle managers have a high level of PA. Finally, The general staff has the characteristics of weak professional ability, less work experience, and weak human communication ability. In addition, patients usually hold a distrustful attitude toward them [40]. Thus, compared with middle and senior managers, the general staff had higher levels of DP and reduced PA. Previous studies have shown that by strengthening the training of professional knowledge and communication skills for the general staff, their professional competence and communication skills can be enhanced [41], and thus their job burnout can be reduced [17].

The job treatment of different positions can be represented the value of an individual’s work. It may be an underlying factor for the EE of middle managers and reduced PA of general staff. The workload and level of difficulty experienced by middle managers are not lower than those of senior managers, but a clear gap between the two groups has been found. This gap leads to their low motivation to work and serious negative emotions. Thus, they have higher levels of EE. The general staff have a heavy workload, but their job treatment is generally low, and thus their sense of personal achievement is low [20]. Adjusting the job treatment of middle managers and general staff not only can increase their enthusiasm to work but also can stabilize the management team.

The career development system is unsound, and this situation may explain the high levels of EE in middle managers and reduced PA in the general staff. First, promotion is usually a step-up from a low-level position to a high-level position. Middle and senior managers constitute a small part of an organization and are thus relatively few. This condition directly limits promotion opportunities for middle managers and general staff [42]. Second, owing to the difficulty in achieving promotion and related requirements, middle managers and the general staff need to undergo strict assessments if they want to be promoted. Middle managers, as backbones of businesses, face increasing pressure for promotion. They have higher levels of EE than senior managers and general staff. Therefore, under the premise of limited opportunities for promotion, the career development system needs to be improved for the stability of management teams [43].

The study findings also indicate that certain individual and job characteristics play significant roles in the burnout experienced by rural primary healthcare doctors. Compared to females, males are more prone to experiencing burnout, possibly due to their strong motivation and sense of achievement. Healthcare professionals aged 40 and above are more susceptible to burnout compared to those below 40, which could be attributed to increased demands in balancing family and work responsibilities, as well as limited career development opportunities. Individuals with higher educational qualifications exhibit higher levels of job burnout, possibly because of their higher expectations for career advancement [44]. On the other hand, healthcare professionals with intermediate professional titles experience greater pressure for promotion compared to those with junior or senior titles; this might be attributed to their strong desire to change their status and achieve success at this career stage [45]. Primary healthcare doctors who work an average of over 50 h per week are most likely to experience job burnout, highlighting the imbalance between the number of primary healthcare workers and their workload, leading to extended working hours for primary healthcare doctors [46]. Additionally, burnout is more severe in western regions compared to eastern and central regions, which may be due to the uneven distribution of medical resources among these areas.

### Limitations

This study has several limitations. First, although the study surveyed each sampled institution, not all personnel responded, and thus the risk of selection bias cannot be ruled out. Nevertheless, a response rate of 83.90% greatly reduced the impact of this selection bias. Second, the different positions represent only where they are located in organizations rather than where they are located in a system as a whole. Finally, this study uses cross-sectional data and cannot determine conclusions about causality. Hence, future in-depth studies should be conducted to explore other factors that may influence burnout in different positions. Despite these limitations, the results of this study can help improve our understanding of the relationship between job characteristics and burnout among healthcare workers in different positions.

### Conclusion

Job burnout is varied by position in health systems. Middle managers showed greater personal stress, and the general staff showed the lowest interpersonal and self-evaluation dimensions of burnout. Job duty, job capability, job treatment, and career advancement in different positions can be the factors for these results. Targeted measures should considered for the reduction of job burnout among different positions, including providing appropriate training and development opportunities, formulating relevant career planning and management strategies, and implementing rational personnel allocation and work design. These measures not only help alleviate individual job burnout but also contribute to improving the performance of the healthcare system. Meanwhile, healthcare professionals are facing increasing work pressure and numerous new challenges due to the impact of significant public health events. Despite some policies encouraging and motivating primary healthcare workers, the relevant policies are still not fully developed, and it is necessary to further refine them to address these challenges. Especially in future research and practice, the influence of the public and policy context on job burnout must be taken into account.
